# A Preliminary Study of the Microbial Resources and Their Biological Activities of the East China Sea

**DOI:** 10.1155/2011/806485

**Published:** 2011-07-19

**Authors:** Xiaoling Lu, Xiaoyu Liu, Cong Long, Guoxiang Wang, Yun Gao, Junhua Liu, Binghua Jiao

**Affiliations:** Department of Biochemistry and Molecular Biology, College of Basic Medical Sciences, Second Military Medical University, Shanghai 200433, China

## Abstract

East China Sea is one of the four sea areas in China, which possesses peculiar ecological environment and many kinds of living creatures, especially the microorganisms. We established the East China Sea microorganism library (during 2006–2010) for the first time, which stored about 30000 strains that covered most kinds of the species. In this paper, 395 pure strains of East China Sea microorganism library which belong to 33 different genera were mainly introduced. *Sulfitobacter*, *Halomonas*, *Bacillus*, *Pseudoalteromonas*, and *Idiomarina* were the most dominant species. On the large-scale biological activity screening of the 395 strains, 100 strains possess different biological activities based on different screening models, of which 11.4% strains have antibacterial activities, 15.9% have cytotoxicity activities, and 6.1% have antioxidation activities. Besides, the secondary metabolites of 6 strains with strong biological activities were studied systematically; diketopiperazines and macrocyclic lactones are the active secondary metabolites. The species and the biological activity of microorganisms diversity, the abundant structure type of the secondary metabolites, and their bioactivities all indicate that East China Sea is a potent marine microorganisms-derived developing resource for drug discovery.

## 1. Introduction

Since the Scottish scientist Alexander Fleming discovered penicillin from the *penicillum* fungi in 1929, bioactive secondary metabolites isolated from the microorganisms have been the abundant resources for the drug discovery. The traditional sources of bioactive compounds were terrestrial microorganisms which could be easily obtained and readily explored. In comparison, the ocean source was scarcely studied. However, since the 1970s, as a consequence of the advanced technology, the ocean has become an attracting area of drug development because of the structural diversity and pharmacological potential which were presented by the novel scaffold molecules isolated from this environment [1]. In 2009, scientists wrote a report that the distribution by phylum for 2006 and 2007 is compared with the historic average derived for the period 1965–2005, the production of marine natural products of microorganisms took a spectacular rise, especially the fungi and bacteria of marine origin [2]. It had also been known that the geographic region of collection sources in output mainly focused on the Caribbean, the China Sea, the Indian Ocean, Japan, and the Western Pacific. The emergence of the China Sea as a significant source of new compounds is particularly obvious.

China is a vast ocean country, which includes four sea areas, such as Bohai Sea, Yellow Sea, South China Sea, and East China Sea. Our country possesses coastline of 18,000 kilometers and the island coastline of 14,000 kilometers, and has the sovereignty and jurisdiction over the sea area of about 3 million square kilometers spanning the tropical zone, subtropical zone, temperate zone, and cold zone. Besides, China Sea possesses four kinds of marine ecosystems, including coastal wetland ecosystems, coral reef ecosystems, upwelling ecosystems, and deep sea ecosystems, which produce many kinds of microorganisms and have abundant resources. The rest three sea areas, except for East China Sea, have been systematically studied [3, 4], while microbial resources of the large-scale survey and the study of the strains with biological activities in the East China Sea have not been reported. 

East China Sea mainly has the upwelling ecosystems and deep sea ecosystems. On one hand, upwelling marine ecosystem possesses higher biological diversity than other ecosystem because of the rich nutrients; on the other hand, some animals and microorganisms with specific structures mainly exist in the deep-sea ecosystem. Overall, the East China Sea has great potential for drug development because of its rich and unique microbial resources. 

In order to discover the bioactive secondary metabolites of the microorganisms from the East China Sea, it is essential to do the large-scale survey of the microbial resources of East China Sea. In this paper, we mainly reported the marine microorganisms, their biological activities such as antimicrobial, antitumor, antioxidant activities, and some secondary metabolites of the strains with biological activities in the East China Sea.

## 2. Materials and Methods

### 2.1. Sampling

The sea water and sea sediment samples were collected in 2006 at a depth of 30 m in the East China Sea (27°15′50′′ N120°45′22′′ E ~ 31°24′7′′ N122°40′48′′ E), and we totally obtained 100 sea sediment sample and 122 sea water sample, which were all stored in refrigerator at −80°C. Weigh 5 g sea sediment, add 45 mL sterile artificial water, shake and mix fully, and stand settlement. Sterile sea water and the supernatant of the sea sediment were progressively diluted with sterilized artificial seawater step by step into 10^−1^, 10^−2^, 10^−3^, 10^−4^, and 10^−5^. Take 0.2 mL every gradient diluent and spread them into different medium plates, incubate at 25°C for 7–14 days, and then pick the single colony according to the differences of the sample and the morphological of the strains. Streak plate method combined with the microscope observation is used during the separation process. All these pure strains were now conserved by freezing at −80°C and at 4°C in our laboratory.

### 2.2. Determination of 16S rRNA Sequence

According to the method described by Rainey et al. [5], the genomic RNAs of all the pure strains were prepared by Genomic RNA Isolation kit (Watson). Then, gene encoding 16S rRNA was amplified by PCR with 16S rRNA Bacterial Identification PCR kit (TaKaRa). An ABI BigDye and Terminator 3.1 cycle sequencing kit (Applied Biosystems) and an automated RNA sequencer (model ABI 3730; Applied Biosystems) were used to determine 16S rRNA gene sequence.

### 2.3. Activity Assays

#### 2.3.1. Assay of Antibacterial Activities

All strains were respectively inoculated in tubes containing 5 mL corresponding liquid medium and cultured on a rotary shaker (150 rpm) at 28°C for 3 ~ 5 days. The fermented broth was filtered through cheese cloth to separate the supernatant and thallus. Antibacterial activity of all the supernatants was evaluated by zone of inhibition using the agar well diffusion assay. The indicator strains are *B. subtilis *(ATCC 6633), *E. coli *(ATCC 25922), and *S. aureus *(ATCC 25923). In this part, ampicillin and blank medium were used as the positive and negative control, respectively.

#### 2.3.2. Assay of Cytotoxic Activities

The entire supernatants of cultured broth were prepared as described above. Cytotoxic activities of these supernatants were evaluated by MTT method [6] using HepG2, HL-60, SMMC 7721, MCF, P388, S180, and SW1990 cell lines. In MTT assay, the cell lines were grown in RPMI-1640 supplemented with 10% fetal bovine serum (FBS) under a humidified atmosphere of 5% CO_2_ and 95% air at 37°C. Cell suspension (90 *μ*L) at a density of 5 × 10^4^ cell/mL was plated in 96-well microtiter plates and incubated for 24 h under the mentioned condition. The supernatants (10 *μ*L) of cultured broth was added to each well and further incubated for additional 72 h in the same condition. Then, 10 *μ*L of MTT solution (5 mg/mL in IPMI-1640 medium) was added to each well and incubated for 4 h. The old medium (110 *μ*L) containing MTT in 96-well microtiter plates was gently replaced by DMSO and pipetted to dissolve any formazan crystals. Absorbance was then determined on a Spectra Max Plus plate reader at 540 nm. In this part, actinomycin D was used as the positive control, normal saline was used as negative control, and 10% DMSO solution was used as blank control.

#### 2.3.3. Assay of Antioxidant Activities

Seed culture (50 mL) of all strains was inoculated into Erlenmeyer flask (2000 mL) containing the liquid medium (500 mL) and cultured on a rotary shaker (150 rpm) at 28°C for 5 days. The fermented whole broth was centrifugated at 12000 rpm for 5 min and separated from supernatant and thallus. The supernatant was extracted with ethyl acetate for 3 times, and the combined extracts were evaporated in vacuum at 30°C to dryness to yield the crude extract, which was dissolved at a concentration of 1 mg/mL in methanol for further analyses.

The DPPH assay was evaluated according to the method described by Hu and Kitts [7] with a little modify. Briefly, crude extract in methanol solution (0.3 mL) mixed with 0.06 mmol/L DPPH (0.5 mL) dissolving in ethanol solution and stood at room temperature in the dark for 20 min when the absorbance at 517 nm was taken. Capacity of free-radical scavenging was evaluated by the inhibition percentage, calculated from the following equation: Inh% = [(A_control_ − A_sample_)/A_control_] × 100, where A_control_ = absorbance of 0.06 mmol/L DPPH (0.5 mL) with methanol (0.3 mL), A_sample_ = absorbance of 0.06 mmol/L DPPH (0.5 mL) with sample solution (0.3 mL).

The ABTS assay was evaluated according to the method of Kajal and Paulraj [8] with a little modify. Briefly, a stock solution of ABTS radical cation was prepared by dissolving ABTS (0.25 mmol/L, 5 mL in water) with potassium persulfate (2 mmol/L, 88 mL). The reaction mixture was diluted to 25 times volume with EtOH and left to stand at room temperature overnight (13 h) in the dark. The ABTS assay was started by mixing the diluted ABTS solution (0.9 mL) with crude extract in methanol solution (0.1 mL) and stood at room temperature in the dark for 20 min when the absorbance at 734 nm was taken. Capacity of free-radical scavenging was evaluated by the inhibition percentage, calculated from the following equation; Inh% = [(A_control_ − A_sample_)/A_control_] × 100, where A_control_ = absorbance of diluted ABTS solution (0.9 mL) with methanol (0.1 mL), A_sample_ = absorbance of diluted ABTS solution (0.9 mL) with sample solution (0.1 mL). Quercetin was used as positive control in the two tests.

### 2.4. Fermentation of the Pure Cultured Strains

The strains F81612, F201721, F8712, F321122, F121122, and M44 have been isolated from sea sediment and sea water of East China Sea, respectively. They were all isolated on F1 medium with incubation at 28°C. F1 Medium: Glucose (6 g), peptone (5 g), and yeast extract (1 g) were dissolved in artificial seawater (1 L). A statistical methodology, combining Plackett-Burman design (PBD) with response surface methodology (RSM), was applied to optimize the biological activity of the strains in the fermentation [9]. Each strain was fermented as follows: 40 2 L Erlenmeyer flasks each containing 700 mL F1 medium (set to the pH 6.5 before sterilization) were inoculated and grown for 4 ~ 7 days at 28°C while shaking at 90 rpm.

### 2.5. Isolation of the Secondary Metabolites

The 6 entire culture broths were all centrifugated at 12000 rpm for 15 minutes, respectively, got the supernatants. The each supernatant was extracted with ethyl acetate for 3 times. The combined extracts were evaporated in vacuum at 30°C to dryness to yield crude extract. Then, the each crude extract was subjected to Sephadex LH-20 and silica gel column, middle-pressure LC with the gradient elution, and chromatographed on preparative HPLC columns repeatedly, which yielded many secondary metabolites.

## 3. Results

### 3.1. Taxonomy of the Studied Pure Strains

The 16S rRNA gene sequence has been determined for a large number of strains. GenBank, the largest databank of nucleotide sequences, has over 20 million deposited sequences, of which over 90,000 are of 16S rRNA gene. This means that there are many previously deposited sequences against which to compare the sequence of an unknown strain [10]. 16S rRNA sequences of 416 pure strains have been determined. After blasting with the GenBank sequences, it is known that a total of 395 strains can be determined their taxonomic status (Table 1). 395 strains belong to 33 different genera, which *Sulfitobacter*, *Halomonas*, *Bacillus*, *Pseudoalteromonas*, and *Idiomarina* were the most dominant species.

### 3.2. Antibacterial Activities

45 strains of the tested pure isolated microorganisms exhibited antibacterial activities, among of which most strains belong to the* Bacillus*, *Sulfitobacter*, and *Pseudoalteromonas* (Table 2). The antibacterial activities of marine *Bacillus* mainly focused on the killing *S.aureus *and *B.subtilis*, while the *Sulfitobacter *mainly showed the strong activities of killing *S.aureus*, and *Pseudoalteromonas* mainly showed the strong activities of killing *E.coli*.

### 3.3. Cytotoxic Activities

The tested pure isolated microorganisms exhibited good cytotoxicity activities to several cell lines, like HepG2, Hela, MCF, and SMMC 7721. It means not all the strains are susceptible to all the tested cell lines, which signifies the differences between the genus and the strains. 63 strains of the tested pure isolated microorganisms exhibited cytotoxicity, among of which most strains were sensitive to the HepG2 cell line and their inhibitory ratio mostly exceed 20% (Table 3). 

**Figure 1:**
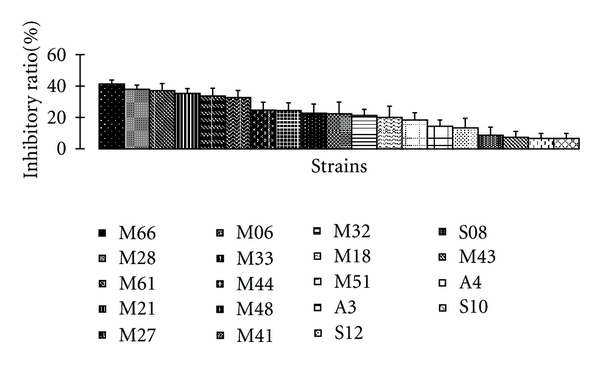
Strains of *Sulfitobacter *sp. with cytotoxicity activities to HepG2 cell line. All the cultured broth of the strains were tested at a fixed concentration 500 *μ*g/mL. Each experiment was repeated 3 times, and the inhibitory ratio was the average of the 3 results.

Most strains of *Sulfitobacter*, *Pseudoalteromonas*, and *Halomonas* exhibited good cytotoxic activities. For example, *Sulfitobacter *sp. is the common species of pure cultured strains. In this species, most strains show good cytotoxic activities to HepG2 cell line (Figure 1). About 25% strains of this species possess strong cytotoxicity activities, whose inhibitory ratios exceed 30%, while the inhibitory ratios of another 30% strains exceed 25%. Thus, it can be seen that *Sulfitobacter *sp. is the potential ones which may produce cytotoxic secondary metabolites.

### 3.4. Antioxidant Activities

**Figure 2:**
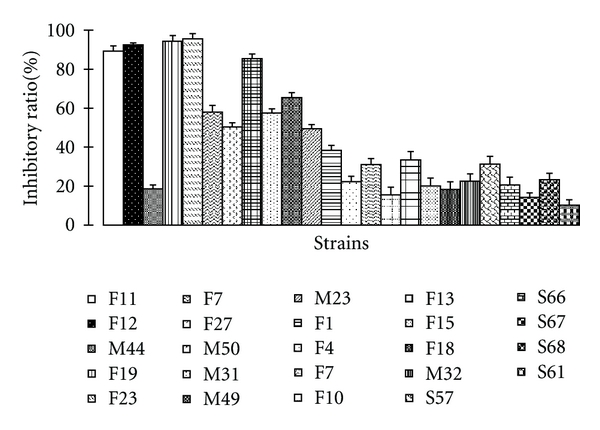
24 strains with best antioxidant activity in DPPH*·* assay. All the cultured broths of the strains were tested at a fixed concentration 375 *μ*g/mL. Each experiment was repeated 3 times, and the inhibitory ratio was the average of the 3 results.

**Figure 3:**
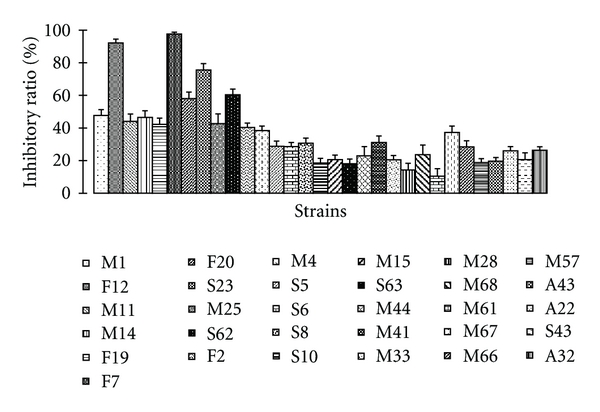
30 strains with best antioxidant activity in ABTS*·*+ assay. All the cultured broths of the strains were tested at a fixed concentration 375 *μ*g/mL. Each experiment was repeated 3 times, and the inhibitory ratio was the average of the 3 results.

The antioxidant activity of the pure cultured strains was determined by the method of spectrophotometry DPPH*·* radical scavenging assay and ABTS*·*+ radical cation decolorization assay. We screened 395 fermented liquid extract of strains, among of which 24 strains show antioxidant activity using the DPPH*·* radical scavenging assay, while 30 strains exhibit antioxidant activity using the ABTS+*·* radical cation decolorization assay (Figures 2 and 3). In the DPPH*·* assay, inhibitory ratio of the 11 strains exceed 40%, and 3 strains exceed 90%. In the ABTS*·*+ assay, inhibitory ratio of the 10 strains exceed 40%, among of which strain F7 has the highest inhibitory ration 98.97%. It can be seen that strains F19, F12, and F23 all exhibit good antioxidant activity in the both methods, which deduce that these 3 strains may have 2 antioxidant mechanisms at least.

### 3.5. The Study of the Secondary Metabolites of the Strains

**Figure 4:**
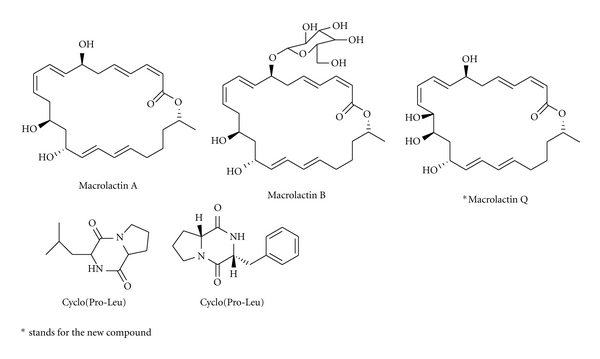
The new secondary metabolites and the compounds with the good biological activities isolated from the strain F201721, F81612, F8712, F321122, F121122, and M44.

In our screening program for bioactive principles from marine microorganisms, we pick up 6 strains (F81612, F201721, F8712, F321122, F121122, and M44) of different genus with strong biological activities to study their bioactive secondary metabolites (Table 4, Figure 4). These 6 strains mainly belong to 3 species which is *Bacillus*, *Pseudoalteromonas*, and *Sulfitobacter*. From the isolated secondary metabolites, it is known that: (1) the main type of the metabolites of these species is cyclopeptide, especially cyclodipeptide, (2) some strains have the common secondary metabolites, for example, macrolactins, cylco(Phe-Ile), cylco(Pro-Ile), and cyclo (Pro-Leu) et al., and (3) the secondary metabolites isolated from different strains signify the different metabolite mechanisms between the genus and the strains.

From the secondary metabolites of our finding, another important kind of the metabolites is macrolide. In 2007, our group found a new macrolactin named macrolactin Q which was isolated from a marine *Bacillus subtilis* [11, 12], and besides, we also isolated macrolactin A and macrolactin B from the same marine *Bacillus subtilis*. Macrolactin Q, macrolactin A and macrolactin B all exhibited antimicrobial activity, macrolactin Q showing stronger antibacterial activity against* E. coli* than *S. aureus* and *B. subtilis*. Macrolactin Q inhibited bacterial growth against *E.coli* with a MIC of 0.2 *μ*g/mL, while inhibiting weaker bacterial growth against *S. aureus* and *B. subtilis* with a MIC of 0.7 *μ*g/mL and 100 *μ*g/mL, respectively.

## 4. Discussion

According to the different sampling points and the different characteristics (morphous, sizes, and colours) of the pure strains, we totally obtained about 30000 pure strains, of which 52% strains belong to the actinomycete, 15% belong to the fungus, and 33% are bacteria. Firstly, we picked up 416 isolated bacteria according to their different morphologic and cultural characteristics to further study their taxonomy, biological activities, and secondary metabolites.

Based on the morphologic and cultural characteristics of the strains, firstly, it is known that majority of them belong to the Gram-negative bacteria. Secondly, more than 85% strains must be cultured in the medium with different concentrations of NaCl, the concentrations of NaCl which range from 2.5% to 15%. Thirdly, it is shown that these strains only need simple nutritional requirements, most of which possess extracellular enzymes and do not use sugar as carbon source and so on. Besides, 395 strains can be determined their taxonomic status, which belong to 33 different genera, and *Sulfitobacter*, *Halomonas*, *Bacillus*,* Pseudoalteromonas*, and* Idiomarina* were the most dominant species.

In the recent years, there are many studies about the biological activities of the secondary metabolites isolated from the marine microorganisms, and these studies mainly focus on the antibacterial, cytotoxic, antioxidation, and antiviral, immunosuppressant activities [13]. In our screening program for bioactive principles from marine microorganisms, it is essential to study the biological activities of the marine microorganisms producing the bioactive secondary metabolites. We mainly studied the activity screening of marine microorganisms referring to the antimicrobial, cytotoxicity, and antioxidant activities.

On the large-scale activity screening of the 395 strains, 100 strains possess different biological activities, of which 45 strains have antibacterial activities, 63 strains have cytotoxicity activities, and 24 strains have antioxidant activities. By this screening, we found that the microorganisms of East China Sea have a wide range of biological activities, including antibacterial activities, cytotoxicity activities, and antioxidant activities, which possess the good potential for the development and utilization. It is good for us to further develop the medicinal microbial use of East China Sea on the foundation of the biological activity screening of the isolated pure strains.

In our screening program for bioactive principles from marine microorganisms, diketopiperazine and macrolide are the two important bioactive secondary metabolites. Diketopiperazines (DKPs), the smallest cyclic peptides, have been isolated from marine microorganisms (bacteria, fungi, and actinomycete) [14–17] or the microorganisms associated with sponge [18–20]. As cyclic peptide derivatives, DKPs have been considered as cell-cell signalling compounds. Some L, L-diketopiperazines have recently been known as quorum-sensing bacterial sensors [21, 22], which are used by Gram-negative bacteria for cell-cell communication and regulating gene expression in response to population density. For example, cyclo (L-Pro-L-Phe) and cyclo (L-Pro-L-Leu) are capable of activating or antagonizing LuxR-mediated quorum-sensing system of bacteria [23, 24]. Therefore, the diketopiperazines, which were produced by many species of the microorganisms, suggest a probable role of these compounds in bacterial-bacterial interaction. 

Besides, DKPs represent an important class of biologically active natural products, which play an important ecological role in antifouling [25, 26], antifungal [27] and antibacterial [16]. For example, cyclo (D-Pro-D-Phe) was previously found in marine bacteria associated with *Pecten maximus* and proved to exhibit bioactivity against *Vibrio anguillarum* [16], and meanwhile, it was also proved to have antifouling activity [28]. Cyclo (L-Pro-L-Phe) and cyclo-(L-Pro-L-Leu), isolated from the bacterium *Alcaligenes faecalis* A72, showed moderate inhibitory activity against *S. aureus *[29]. Therefore, the diketopiperazines, which were isolated from the microorganisms of East China Sea, may show many kinds of biological activities, which indicated the biological activities of the microorganisms.

Macrolactins are a group of 24-membered lactones with potent antibacterial or other activities, most of which were derived from the second metabolites of the marine microorganisms, while several of them were also produced by some soil microorganisms as well. There were total 22 isolated macrolactins reported, since the first one was isolated in 1989. Our group found a new macrolactin named macrolactin Q, which possesses strong antibacterial activity against* E. coli* than *S. aureus*

## 5. Conclusion

The East China Sea marine microorganism library (during 2006–2010) was established for the first time, which stored about 30000 strains that covered most kinds of the species. In this paper, 395 strains were mainly described, which belong to 33 different genera. *Sulfitobacter*, *Halomonas*, *Bacillus*,* Pseudoalteromonas*, and* Idiomarina* were the most dominant species, which may signify the genera diversity of the microorganisms of East China Sea. According to the results, 25.3% strains possess different biological activities, like antibacterial, cytotoxicity, and antioxidant activities. The diversity of the species of microorganisms, the biological activities of their secondary metabolites, and the abundant structure type of the secondary metabolites all indicate that East China Sea is a potent microorganism-derived medical resource for drug development.

##  Conflict of Interests

The authors declare that there is no conflict of interests.

## Figures and Tables

**Table 1:** Taxonomy of 395 strains.

Genus	No.	Genus	No.	Genus	No.
*Agrococcus*	8	*Alcanivorax*	3	*Alphaproteobacterium*	4
*Bacillus*	30	*Brachybacterium*	7	*Brevibacterium*	6
*Dietzia*	2	*Erythrobacter*	1	*Exiguobacterium*	10
*Gamma proteobacterium*	5	*Halomonas*	42	*Idiomarina*	36
*Kocuria*	9	*Kurthia*	2	*Loktanella*	3
*Lysobacter*	2	*Marinobacter*	4	*Marinococcus*	3
*Methylarcula*	5	*Micrococcus*	1	*Oceanicola*	4
*Paracoccus*	6	*Photobacterium*	7	*Planococcus*	5
*Pseudoalteromonas*	69	*Pseudomonas*	12	*Psychrobacter*	2
*Rheinheimera*	5	*Salegentibacter*	2	*Staphylococcus*	10
*Sulfitobacter*	62	*Vibrio*	8	*Virgibacillus*	20

**Table 2:** Strains with strongest antibacterial activities.

Strain no.	Genus	*E. coli*	*B. subtilis*	*S. aureus*
ZJ8112	*Pseudoalteromonas*	++	−	−
F121112	*Pseudoalteromonas*	+	−	−
F8712	*Halomonas*	−	−	−
F321122	*Halomonas*	−	−	−
201721	*Pseudoalteromonas*	++	−	+
XB16	*Idiomarina*	−	−	−
MZ0306A2	*Bacillus*	+	+	++
MZ0208A2	*Bacillus*	−	+	++
MZ0204A3	*Bacillus*	−	+	+
P3001	*Bacillus*	−	+	+++
P4004A	*Bacillus*	−	+	+
P16006B	*Idiomarina*	−	+++	−
P3009B	*Idiomarina*	−	+++	−
P16011A	*Bacillus*	−	+	−
P16010C	*Halomonas*	−	++	−
P4004C	*Bacillus*	−	++	++
XF22−2	*Sulfitobacter*	−	−	++
P4003B	*Sulfitobacter*	−	−	+
P12012B	*Sulfitobacter*	−	−	++

Note: “+++”means the diameters of the inhibition zone exceeding 15 mm; “++” means the diameters of the inhibition zone 10–15 mm; “+” means the diameter of the inhibition zone exceeding 6–10 mm; “−”means no antibacterial activities.

**Table 3:** Strains with strongest cytotoxic activities of the 395 strains.

Strain no.	Genus	Sensitive tumor strain	Inhibitory ratio
F321121	*Halomonas*	SMMC 7721	17.6%
P12002	*Sulfitobacter*	HeLa, MCF	35%, 12%
F81612	*Bacillus*	HeLa, SW1990	39%, 9.3%
F81611	*Bacillus*	HeLa	29.1%
P3001	*Vibrio*	HepG2	28%
P3002	*Rheinheimera*	HepG2, MCF	27%
P16006A	*Kocuria*	HepG2	29%
P16006B	*Idiomarina*	HepG2	32%
P16001	*Halomonas*	HeLa, MCF	18.4%, 20.4%
P4003B	*Sulfitobacter*	HepG2, P388	31%, 7.9%
P12006A2	*Sulfitobacter*	HepG2	31%
P4006B	*Marinobacter*	HepG2, P388	42%, 13%
MZ0206A2	*Bacillus*	SMMC7721, S180	11%, 19%
MZ0208A2	*Bacillus*	SMMC 7721	21%
P16003	*Kocuria*	HepG2	23%
P4004B	*Idiomarina*	HepG2	31%
P12002	*Idiomarina*	HepG2	28.7%
P12012B	*Sulfitobacter*	HepG2, SW1990	14.9%, 17%
P12004D	*Sulfitobacter*	HepG2, S180	16.7%, 23%

All the cultured broths of the strains were tested at a fixed concentration 500 *μ*g/mL. Each experiment was repeated 3 times, and the inhibitory ratio was the average of the 3 results.

**Table 4:** Overview of the 6 strains with biological activities.

No.	Genus	Biological activity	Main type of the secondary metabolites
F81612	*Bacillus*	Antibacterial	cyclopeptide, indole derivatives, and macrolide
F201721	*Bacillus*	Antibacterial	cyclopeptide, indole derivatives, and macrolide
F8712	*Pseudoalteromonas*	Cytotoxicity	cyclopeptide
F321122	*Pseudoalteromonas*	Cytotoxicity	cyclopeptide and indole derivatives
F121122	*Sulfitobacter*	Cytotoxicity	cyclopeptide
M44	*Sulfitobacter*	Cytotoxicity	cyclopeptide

## Abbreviations

DPPH: Diphenyl-1-picrylhydrazyl
ABTS*·*+: 2,2′-azino-bis (3-ethylbenzthiazoline-6-sulphonic acid)
DKPs: Diketopiperazines
MIC: Minimal inhibitory concentration.

## References

[1] Faulkner DJ (1999). Marine natural products. *Natural Product Reports*.

[2] Blunt JW, Copp BR, Hu WP, Munro MHG, Northcote PT, Prinsep MR (2009). Marine natural products. *Natural Product Reports*.

[3] Wang S (2001). Study of marine microbial resources in Huanghai, Bohai and Liaoning offshore of China. *Journal of Jinzhou Normal College*.

[4] Sun F, Wang B, Li G (2008). Diversity of bacteria isolated from the South China Sea sediments. *Acta Microbiologica Sinica*.

[5] Rainey FA, Ward-Rainey N, Kroppenstedt RM, Stackebrandt E (1996). The genus Nocardiopsis represents a phylogenetically coherent taxon and a distinct actinomycete lineage: proposal of nocardiopsaceae fam. nov. *International Journal of Systematic Bacteriology*.

[6] Wang FZ, Fang Y, Zhu T (2008). Seven new prenylated indole diketopiperazine alkaloids from holothurian-derived fungus *Aspergillus fumigatus*. *Tetrahedron*.

[7] Hu C, Kitts DD (2000). Studies on the antioxidant activity of echinacea root extract. *Journal of Agricultural and Food Chemistry*.

[8] Kajal C, Paulraj R (2010). Sesquiterpenoids with free-radical-scavenging properties from marine macroalga Ulva fasciata Delile. *Food Chemistry*.

[9] Chen D, Han Y, Gu Z (2006). Application of statistical methodology to the optimization of fermentative medium for carotenoids production by Rhodobacter sphaeroides. *Process Biochemistry*.

[10] Stan-Lotter H, Pfaffenhuemer M, Legat A, Busse HJ, Radax C, Gruber C (2002). Halococcus dombrowskii sp. nov., an archaeal isolate from a Permian alpine salt deposit. *International Journal of Systematic and Evolutionary Microbiology*.

[11] Lu XL (2008). Marine drugs—macrolactins. *Chemistry & Biodiversity*.

[12] Lu XL, Xu QZ, Shen YH (2008). Macrolactin S, a novel macrolactin antibiotic from marine Bacillus sp. *Natural Product Research*.

[13] Vaishnav P, Demain AL (2011). Unexpected applications of secondary metabolites. *Biotechnology Advances*.

[14] Adamczeski M, Reed AR, Crews P (1995). New and known diketopiperazines from the Caribbean sponge, Calyx cf. podatypa. *Journal of Natural Products*.

[15] De Rosa S, Mitova M, Tommonaro G (2003). Marine bacteria associated with sponge as source of cyclic peptides. *Biomolecular Engineering*.

[16] Fdhila F, Vazquez V, Sánchez JL, Riguera R (2003). DD-diketopiperazines: antibiotics active against Vibrio anguillarum isolated from marine bacteria associated with cultures of Pecten maximus. *Journal of Natural Products*.

[17] Ovenden SPB, Sberna G, Tait RM (2004). A diketopiperazine dimer from a marine-derived isolate of *Aspergillus niger*. *Journal of Natural Products*.

[18] Fu X, Ferreira MLG, Schmitz FJ, Kelly-Borges M (1998). New diketopiperazines from the sponge *Dysidea chlorea*. *Journal of Natural Products*.

[19] Fu X, Zeng LM, Su JY, Pais M (1997). A new diketopiperazine derivative from the South China Sea sponge *Dysidea fragilis*. *Journal of Natural Products*.

[20] Vergne C, Boury-Esnault N, Perez T (2006). Verpacamides A-D, a sequence of C_11_N_5_ diketopiperazines relating cyclo(Pro-Pro) to cyclo(Pro-Arg), from the marine sponge *Axinella vaceleti*: possible biogenetic precursors of pyrrole-2- aminoimidazole alkaloids. *Organic Letters*.

[21] De Kievit TR, Iglewski BH (2000). Bacterial quorum sensing in pathogenic relationships. *Infection and Immunity*.

[22] De Rosa S, Mitova M, Tommonaro G (2003). Marine bacteria associated with sponge as source of cyclic peptides. *Biomolecular Engineering*.

[23] Degrassi G, Aguilar C, Bosco M, Zahariev S, Pongor S, Venturi V (2002). Plant growth-promoting *Pseudomonas putida* WCS358 produces and secretes four cyclic dipeptides: cross-talk with quorum sensing bacterial sensors. *Current Microbiology*.

[24] Holden MTG, Chhabra SR, De Nys R (1999). Quorum-sensing cross talk: isolation and chemical characterization of cyclic dipeptides from *Axinella vaceleti* and other Gram-negative bacteria. *Molecular Microbiology*.

[25] Wang Y, Mueller UG, Clardy J (1999). Antifungal diketopiperazines from symbiotic fungus of fungus-growing ant *Cyphomyrmex minutus*. *Journal of Chemical Ecology*.

[26] Li ZY, Liu Y (2006). Marine sponge *Craniella austrialiensis*-associated bacterial diversity revelation based on 16S rDNA library and biologically active actinomycetes screening, phylogenetic analysis. *Letters in Applied Microbiology*.

[27] Musetti R, Polizzotto R, Vecchione A (2007). Antifungal activity of diketopiperazines extracted from *Alternaria alternata* against *Plasmopara viticola*: an ultrastructural study. *Micron*.

[28] Yang LH, Miao L, Lee OO (2007). Effect of culture conditions on antifouling compound production of a sponge-associated fungus. *Applied Microbiology and Biotechnology*.

[29] Li ZY, Peng C, Shen Y, Miao X, Zhang H, Lin H (2008). L,L-diketopiperazines from *Alcaligenes faecalis* A72 associated with South China Sea sponge *Stelletta tenuis*. *Biochemical Systematics and Ecology*.

